# Forward genetic screens identify a role for the mitochondrial HER2 in *E*-2-hexenal responsiveness

**DOI:** 10.1007/s11103-017-0659-8

**Published:** 2017-09-16

**Authors:** Alessandra Scala, Rossana Mirabella, Joachim Goedhart, Michel de Vries, Michel A. Haring, Robert C. Schuurink

**Affiliations:** 1 Department of Plant Physiology, Swammerdam Institute for Life Sciences, Science Park 904, 1098 XH Amsterdam, The Netherlands; 2 Department of Molecular Cytology, Swammerdam Institute for Life Sciences, Science Park 904, 1098 XH Amsterdam, The Netherlands

**Keywords:** *Arabidopsis*, *E*-2-hexenal, Mitochondria, Oxidoreductase, Redox status

## Abstract

**Key message:**

This work adds a new player, HER2, downstream of the perception of *E-2*-hexenal, a green leaf volatile, and shows that *E-2*-hexenal specifically changes the redox status of the mitochondria.

**Abstract:**

It is widely accepted that plants produce and respond to green leaf volatiles (GLVs), but the molecular components involved in transducing their perception are largely unknown. The GLV *E*-2-hexenal inhibits root elongation in seedlings and, using this phenotype, we isolated *E*-2-hexenal response (*her*) *Arabidopsis thaliana* mutants. Using map-based cloning we positioned the *her2* mutation to the At5g63620 locus, resulting in a phenylalanine instead of serine on position 223. Knockdown and overexpression lines of *HER2* confirmed the role of *HER2*, which encodes an oxidoreductase, in the responsiveness to *E*-2-hexenal. Since *E*-2-hexenal is a reactive electrophile species, which are known to influence the redox status of cells, we utilized redox sensitive GFP2 (roGFP2) to determine the redox status of *E*-2-hexenal-treated root cells. Since the signal peptide of HER2 directed mCherry to the mitochondria, we targeted the expression of roGFP2 to this organelle besides the cytosol. *E*-2-hexenal specifically induced a change in the redox status in the mitochondria. We did not see a difference in the redox status in *her2* compared to wild-type *Arabidopsis*. Still, the mitochondrial redox status did not change with *Z-*3-hexenol, another abundant GLV. These results indicate that HER2 is involved in transducing the perception of *E*-2-hexenal, which changes the redox status of the mitochondria.

**Electronic supplementary material:**

The online version of this article (doi:10.1007/s11103-017-0659-8) contains supplementary material, which is available to authorized users.

## Introduction

Green leaf volatiles are C_6_ volatiles, which are produced by plants in response to herbivory, wounding and environmental stress (Croft et al. 1993; Fall et al. 1999; Gouinguené and Turlings 2002; Heiden et al. 2003; Shiojiri et al. 2000, 2006; Turlings et al. 1995). Biosynthesis of these volatiles occurs mainly from α-linolenic acid by the sequential action of two enzymes, a lipoxygenase (LOX), which dioxygenates at the C_13_ position, and hydroperoxide lyase (HPL; Hatanaka 1993; Matsui 2006; Matsui et al. 2000; Mochizuki et al. 2016), followed by the activity of an aldehyde dehydrogenase (ADH), an isomerase (Kunishima et al. 2016) and acetyltransferase (D’Auria et al. 2007) in order to produce the GLV bouquet of C_6_-aldehydes, alcohols and their esters.

Plants have the ability to sense GLVs although the mechanism of the perception remains unknown (Matsui 2016; Scala et al. 2013). Since plants respond to GLVs with transcriptional changes related to wound and herbivore-induced defenses (Scala et al. 2013), GLVs are now also considered as damaged associated molecular patterns or DAMPs, probably carrying specific information about the attacker (Duran-Flores and Heil 2016). Many responses in plants to GLVs have been described (see e.g. (Scala et al. 2013)) and several of the early signaling events and downstream molecular players have been elucidated. These include plasma membrane depolarization (Zebelo et al. 2012), increases in cytosolic Calcium (Asai et al. 2009; Zebelo et al. 2012) and increased transcript levels of several genes involved in Calcium signaling, including Calmodulin, a Ca^2+^-binding protein (Engelberth et al. 2013). *E*-2-hexenal has been shown to induce abiotic-related transcription factors (Yamauchi et al. 2015) as well as two WRKY transcription factors (Mirabella et al. 2015). WRKY6 negatively regulates glutamate decarboxylase that converts glutamate to γ-amino butyric acid (GABA), a metabolite implicated in the responsiveness to *E*-2-hexenal (Mirabella et al. 2008). In the monocot maize, GLVs can, beside changing the transcriptome (Engelberth et al. 2013), induce the production of jasmonic acid (Engelberth et al. 2004). Plants have also developed a repertoire of enzymatic reactions to modify GLVs upon perception (Matsui 2016), reducing them (Matsui et al. 2012), converting them to glycosides (Sugimoto et al. 2014, 2015) or glutathione conjugates (Davoine et al. 2006; Mirabella et al. 2008).

We set out to find additional molecular players that mediate responses to GLVs using forward genetics. To reduce the complexity of this study we decided to focus on *E*-2-hexenal, which has an α,β−unsatured carbonyl moiety with a high reactivity towards nucleophils, such as the thiol- or amino-group (Esterbauer et al. 1991; Farmer and Davoine 2007), giving it the denomination as a reactive electrophile species (RES) (Farmer and Davoine 2007; Farmer and Mueller 2013; Park et al. 2010). Previously we have shown that the roots of *Arabidopsis* seedlings, when exposed to sub-micromolar concentrations of *E*-2-hexenal, stop growing, similar as with jasmonic acid (Staswick et al. 1992), and that this response is, among the GLVs, specific for *E*-2-hexenal. With this readout we could identify mutants of which the roots grew faster upon *E*-2-hexenal exposure than the wildtype, which we called *E*-2-hexenal response (*her*) mutants (Mirabella et al. 2008). This lead to the characterization of the *her1* mutant and the discovery of the role of GABA downstream of *E*-2-hexenal. We have now used map-based cloning to position the mutation in *her2* to the At5g63620 locus. *HER2* is predicted to encode an oxidoreductase and since RES can influence the redox status of cells (Farmer and Davoine 2007; Mueller and Berger 2009) we studied the effect of *E*-2-hexenal on the redox status of the root cells. Our results put HER2 as a new molecular player in the signaling events upon perception of *E*-2-hexenal and show that *E*-2-hexenal changes the redox status of the mitochondria.

## Materials and methods

### Mutant screen and mapping

The *Arabidopsis* mutant seed population was provided by Dr G-J. de Boer (enzazaden.nl). In brief, *Arabidopsis* seeds, ecotype Col-0, were mutagenized by soaking about 50,000 seeds in 0.2% (v/v) EMS, as previously described by Weigel and Glazebrook (2006). M2 seeds were collected from pools of 1000 plants. For the mutant screen, approximately 35,000 M2 seeds were screened from 12 independent pools as described previously (Mirabella et al. 2008). The *her2* mutant was outcrossed to wildtype ecotype *L*er. F1 plants were allowed to self-fertilize, and the F2 progeny was screened for sustained root growth in the presence of aerial 0.3 µM *E*-2-hexenal. Mapping was essentially done as previously described (Mirabella et al. 2008), with different markers (see Fig. S1 in Supplementary Material).

### Plant lines

SALK-generated T-DNA insertion lines 072101 and 079558 were obtained from the Arabidopsis Biological Resource Center (ABRC, Ohio State University, Columbus, OH, USA (Alonso et al. 2003). For *HER2* overexpressing lines (HER2:OE), the HER2 cDNA was cloned in pGreen0229 under control of the 35S promoter. Col-0 plants were transformed with *Agrobacterium tumefaciens* GV3101 carrying this plasmid and primary transformants were selected on BASTA (50 µg ml^−1^), and in the next generations till homozygous lines were obtained. For redox sensitive GFP lines, Col-0 and *her2* plants were transformed with *A. tumefaciens* GV3101 carrying the plasmid pBinCM-SHMT-roGFP2-GRX1 for expression of roGFP2-GRX1 in mitochondria or pBinCM-roGFP2-GRX1 for cytosolic expression both under control of the *Ubi10* promoter (Jiang et al. 2006; Meyer and Brach 2009; Meyer et al. 2007). Selection was done on kanamycin (15 mg ml^−1^) till homozygous lines were obtained.

### Quantitative RT-PCR

For analysis of transcript levels, total RNA was isolated using Trizol from ten different plants, in three independent experiments and treated with TurBo DNAse (Ambion^®^, http://www.lifetechnologies.com/nl/en/home/brands/ambion.html) to remove DNA. cDNA was synthesized from 1 µg of total RNA using M-MuLV reverse transcriptase (Fermentas, thermoscientificbio.com/fermentas), as described by the manufacturer, in a 20-µl reaction that was diluted to 50 µl prior to using it for the real-time PCR. This was performed in a 20-µl volume containing 2 µl of cDNA, 0.4 pmol of specific primer sets for each gene and 10 µl of Taq™ SYBR Green Supermix with ROX (Bio-Rad, bio-rad.com). PCR conditions were as follows: 95 ℃ for 2 min 30 s (first cycle), 95 °C for 15 s and 60 °C for 30 s (40 cycles). To ensure amplification of a single product during the qRT-PCR reactions, a dissociation protocol was performed in which samples were slowly heated from 55 to 95 °C. qRT-PCR was performed using the ABI Prism 7000 real-time PCR detection system (Applied Biosystems, appliedbiosystems.com) and the data were collected using software (ABI 7000 SDS version 1) provided by the supplier. Transcript levels were normalized to the transcript levels of the *SAND* gene (At2g28390; (Hong et al. 2010)) and quantification was performed as described by (Pfaffl 2001). Primer sequences for HER2 were qHER2fw TGGAGGAAGAGCAAGACAGG, qHER2rev GAGACTGCGTTTGTGA GATTG.

### Volatile treatments

Seeds were surface sterilized and planted into horizontally slit cuts in 1.5% agar plates containing half-strength MS salts, pH 5.8. In this way, roots emerging from the germinated seeds grew on the surface and did not penetrate in the agar. The plates were chilled for 2 days at 4 °C before being placed vertically in an environmental growth chamber with a light (100 µE sec^−1^ m^−2^)/dark cycle of 16/8 h at 21 °C and 70% relative humidity. Plants were grown for 4 days under these conditions before being exposed to volatiles. For the volatile treatment, two Petri dishes, with the lid removed, were placed vertically into airtight glass desiccator (22 l). Technical replicates were done in different desiccators. *E*-2-hexenal was diluted in methanol, and 50 µl of the diluted solutions were applied to a sterile cotton swab, placed in an 50 ml Erlenmeyer flask, between the plates in the desiccators (Mirabella et al. 2015). For the control treatment only methanol (MeOH) was applied. For instance, for a concentration of 0.3 μM aerial *E*-2-hexenal 6.6 μmol was added to the cotton swap in a desiccator of 22 l, but only 7.3% of the *E*-2-hexenal could be recovered from the desiccator (Mirabella et al. 2015). Plates were incubated in the desiccators with the volatiles for 24 h for root length measurement, and were subsequently removed and placed vertically under the growth conditions described above, and root growth was measured 3 days later. For each treatment, the root length of at least ten seedlings for each plant line, distributed over eight plates, was determined with ImageJ software (http://rsbweb.nih.gov/ij/). All experiments were done at least three times. For FLIM measurements plants were treated with 0.13 µM *E*-2-hexenal, *Z*-3-hexenol or methanol as control for 1 h. For each measurement at least eight seedlings per plant line, distributed over four different plates were used. All experiments were done at least three times.

### Confocal and fluorescence lifetime imaging microscopy (FLIM) measurements

Protoplast transfection was done as reported in (Vermeer et al. 2004) and confocal microscopy was performed as published by (Vermeer et al. 2009). Briefly, leaf protoplasts of an *Arabidopsis* line expressing a mitochondrion localized GFP (Nelson et al. 2007) were transfected with a plasmid expressing a fusion between the predicted signal peptide of HER2 (the first 55 aa) and mCherry (pMON999-35S-SP-mCherry).

Fluorescence lifetime imaging was performed using the wide-field frequency domain approach on a home-build instrument (van Munster and Gadella 2005) using a RF-modulated image intensifier (Lambert Instruments II18MD) coupled to a CCD camera (Photometrics HQ) as detector. A 40× objective (Plan NeoFluar NA 1.3 oil) was used for all measurements. The modulation frequency was set to 75.1 MHz. Eighteen phase images with an exposure time of 20–80 ms were acquired in a random recording order to minimize artifacts due to photobleaching (van Munster and Gadella 2004). An argon-ion laser was used for excitation at 488 nm, passed onto the sample by a 495 nm dichroic mirror and emission light was filtered by a 515/30 nm emission filter. For the control experiment, of each plant line, at least eight (4 day-old) seedlings were treated with 20 mM H_2_O_2_ for 30 min (Wierer et al. 2012).

## Results

### The mutation in *her2* maps to At5g63620

**Fig. 1:**
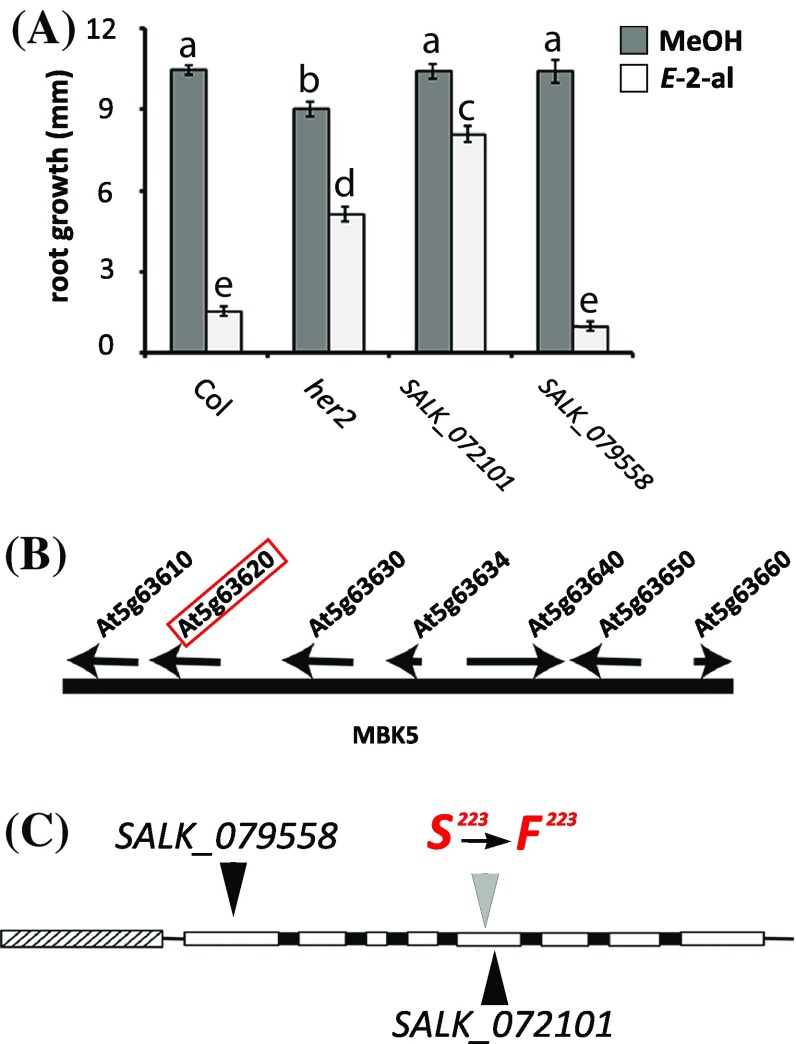
Positional mapping of *her2* location and response to *E*-2-hexenal. **a** Three-day-old seedlings from SALK lines 072101 and 079558, *her2* and wild-type Col-0 were exposed for 24 h to MeOH (as a control as *E*-2-hexenal is dissolved in MeOH) or to 0.3 µM aerial *E*-2-hexenal. Root growth was measured 3 days after the treatment. Values are the mean root length of at least 15 plants, distributed over three plates (± SEM). Different letters above the bars indicate significant differences at a level of P ≤ 0.05, after applying ANOVA followed by a LSD posthoc test. **b** Representation of chromosome 5 between nucleotides 25,464,351 and 25,487,847. Arrows denote the position of the genes present in this area. The red box indicates the At5g63620 locus. **c** Representation of the *HER2* genomic region. Exons (white boxes) introns (black boxes) and putative promoter (grey arched box) are shown. Black triangles indicate T-DNA insertions for the Col-0 SALK_072101 (5th Exon) and SALK_079558 (1st Exon) lines. Amino acid 223 (S, serine) is mutated to a phenylalanine (F) in *her2*

In a previous paper (Mirabella et al. 2008) we described the isolation of different *hexenal response (her*) mutants and we showed that the *her1* mutation was in the GABA transaminase gene (At3g22200). Here we describe the mapping of the mutation in *her2*. The *her2* mutant has a similar phenotype as the *her1* mutant (Mirabella et al. 2008), i.e. the growth of the root of young seedlings is less inhibited by 0.3 μM aerial *E*-2-hexenal than the wild type Arabidopsis ecotype Col-0 of which the root stops growing (Fig. 1a).

Since the trait was segregating as single recessive mutation, we used positional cloning to map the *her2* locus as we did for *her1* (Mirabella et al. 2008), based on the methods developed by Lukowitz et al. (2000) and Jander et al. (2002). We created an F2 population from a cross between *Ler* and *her2* and phenotyped 512 F2 plants. Bulk segregant analysis on 50 plants, with the wt or *her2* phenotype, using the markers developed by (Lukowitz et al. 2000) put the mutation on the lower arm of chromosome 5 (ciw9 marker, data not shown). To further delimit the position of the *her2* locus, all 512 phenotyped F2 plants were genotyped with markers in this area (see Table S1 in Supplementary Material). For markers Mbk5c7 and Mbk5c8, spanning a region of 25 kbp, only three recombinants were identified (see Fig. S1 in Supplementary Material). This interval contains seven genes (Fig. 1b) that were subsequently all sequenced. Only At5g63620 had a point mutation in an exon compared to the published Col-0 genomic sequence (http://www.arabidopsis.org). This point mutation leads to a single amino acid substitution in the predicted At5g63620 protein, from serine (S223) to phenylalanine (F223) (Fig. 1c). According to the conserved domain detection by NCBI (http://ncbi.nlm.nih.gov), amino acid 223 is in a Zn-binding site. The aromatic group of Phenylalanine might thus lead to steric hinder and possibly influences Zn binding.

In order to corroborate the correlation between the genotype of *her2*, i.e. the point mutation in At5g63620, and its phenotype, we tested two SALK lines with T-DNA insertions in At5g63620 for their response to *E*-2-hexenal. SALK line 072101 has a T-DNA insertion in the fifth exon and SALK line 079558 in the first exon of At5g63620 (Fig. 1c). The extent of resistance to *E*-2-hexenal-induced root growth inhibition shown in the *her2* mutant is also displayed in the SALK_072101 mutant (Fig. 1a), even to an higher extent than in *her2*, whereas the SALK_079558 mutant had a similar response as Col-0. The phenotype of the SALK lines correlated with the transcript levels of At5g63620 in these lines: SALK_072101 had significantly reduced transcript levels but SALK_079558 did not (see Fig. S2 in supplementary Material). We thus concluded that the mutation in At5g63620 indeed caused the phenotype of *her2* and refer further to this locus as *HER2*.

### HER2 overexpression leads to higher sensitivity to *E-2*-hexenal

**Fig. 2:**
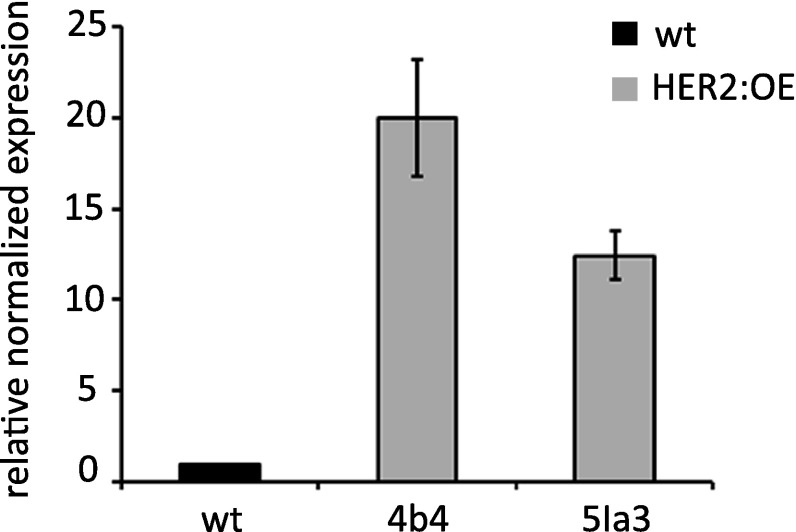
*HER2* transcript levels in *HER2* overexpressing plants. *HER2* transcript levels were measured by qRT-PCR in two independent overexpressing lines (HER2:OE), 4b4 and 5Ia3, and normalized for *SAND* transcript levels. The mean (and min/max) of two independent experiments is presented with the value of the wildtype (wt) set to one

**Fig. 3:**
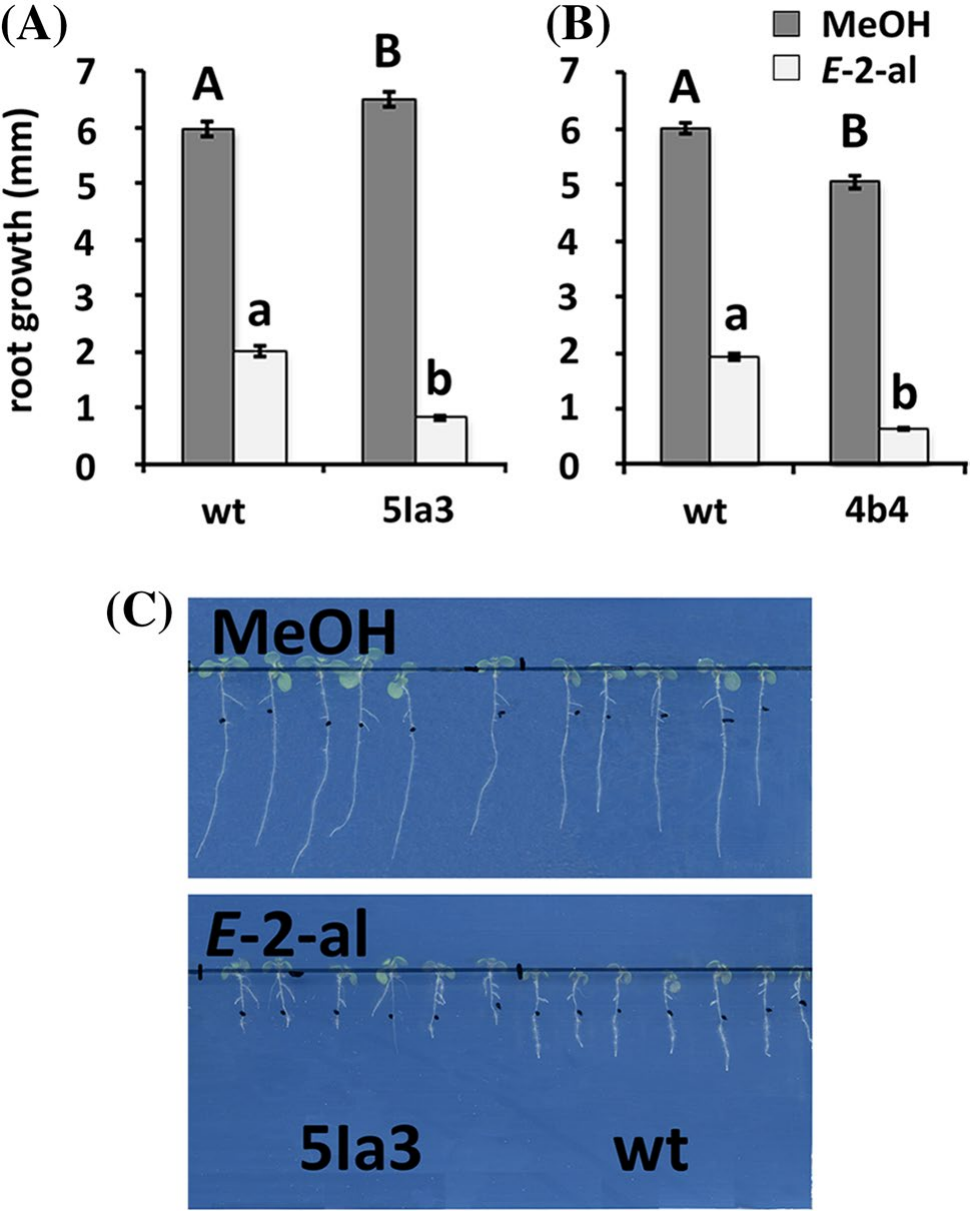
Root growth of 35S-HER2 (4b4 and 5Ia3) and wildtype (wt) seedlings after 24 h treatment with MeOH, or with 0.15 µM (**a** and **c**) or 0.2 µM (**b**) aerial *E*-2-hexenal. The measurements were made 4 days after treatment on at least 30 seedlings (n = 2). Bars represent the means (± SEM). Bars annotated with different letters indicate significant differences between MeOH or *E*-2-hexenal treatment (P < 0.05, ANOVA followed by least significant difference (LSD) post hoc test). **c** Root growth of wild-type (Col-0) and HER2 overexpression (line 5la3) seedlings. The images were taken 4 days after treatment

In order to corroborate the role of At5g63620 (HER2) in the *E*-2-hexenal response further, we overexpressed *HER2* under control of the 35S promoter in Arabidopsis. We characterized two independent lines that had higher *HER2* expression, with somewhat higher transcript levels in line 4b4 than in 5Ia3 (Fig. 2). Four day-old seedlings of these two independent HER2 overexpressing lines (HER2:OE) were subsequently exposed for 24 h to *E*-2-hexenal. We had to use two different *E*-2-hexenal concentrations, 0.2 and 0.15 µM, since the two independent *HER2* overexpression lines were not only more responsive to the aldehyde but also responded differently. Indeed line 4b4 showed a root growth inhibition phenotype at 0.2 µM while the line 5Ia3 did at a lower dose (0.15 µM). In both cases the *HER2* overexpression lines showed a stronger growth inhibition of the roots than the wt (Fig. 3a, b). This confirms the role of HER2 in the responsiveness to *E*-2-hexenal.

### HER2 protein localizes in the mitochondria

**Fig. 4:**
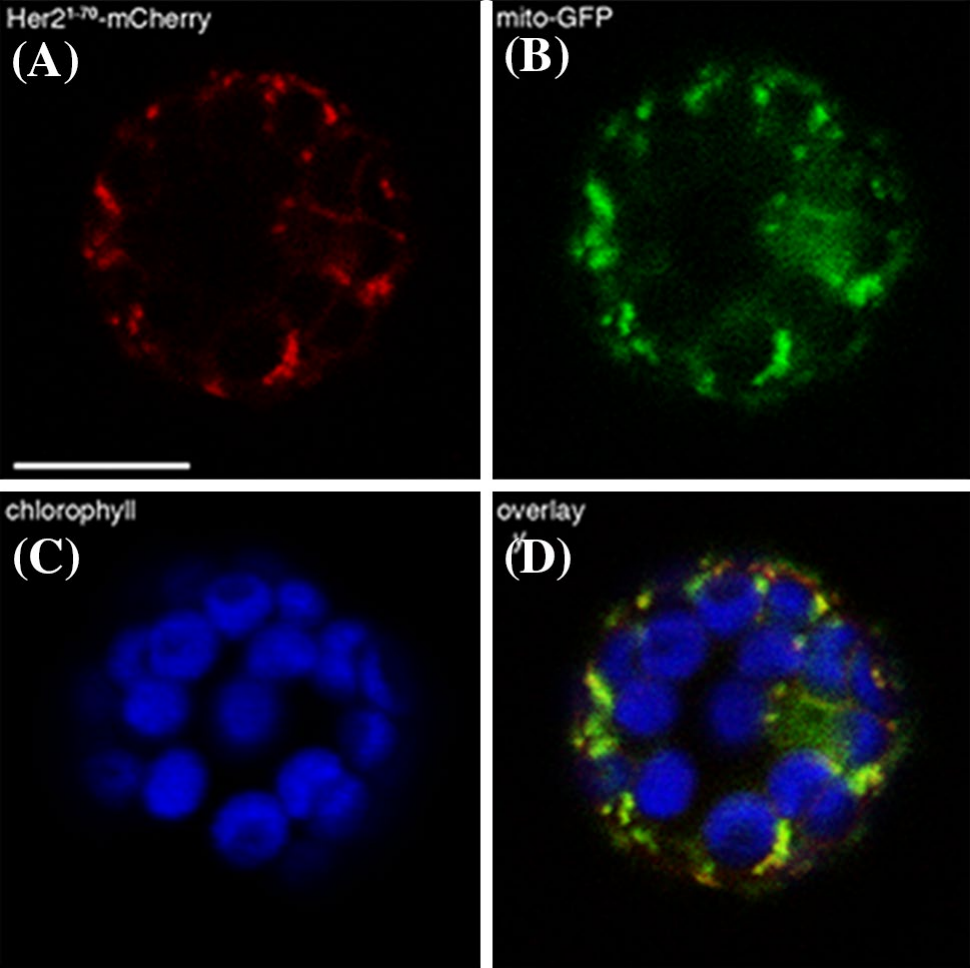
HER2 is localized in the mitochondria. Confocal image of transfected protoplast showing: **a** HER2 signal peptide-mCherry, **b** Mito-GFP reporter, **c** chlorophyll autofluorescence and **d** overlay

The HER2 protein is predicted to be localized in the mitochondrion (http://www.arabidopsis.org). To determine the subcellular localization of HER2 *in planta* we made a construct of the putative signal peptide of HER2 (Wolf PSort, http://www.genscript.com/psort/wolf_psort.html), comprising the first 55 amino acids, fused to mCherry driven by the 35S promoter. We transfected *Arabidopsis* mesophyll protoplasts with this construct (Fig. 4a) expressing a mitochondrial reporter, 35S-mito-GFP (Fig. 4b) (Nelson et al. 2007). The obtained fluorescent signals overlapped (Fig. 4d) indicating that HER2 localizes indeed in the mitochondria. The chlorophyll autofluorescence (blue) is also shown (Fig. 4c) to illustrate the chloroplasts.

### Phylogenetic analysis of HER2

The TAIR database (http://www.arabidopsis.org) predicts that the At5g63620 gene encodes a GroES-like zinc-binding alcohol dehydrogenase family protein. Alcohol dehydrogenases (ADH, alcohol:NAD + oxidoreductase, EC 1.1.1.1) are Zn-binding enzymes that act as dimers and use NAD(P) as co-factor to convert short linear alcohols in their related aldehydes (e.g. ethanol in acetaldehyde) or NAD(P)H for the reciprocal reaction. ADHs are members of the medium-length dehydrogenase/reductase (MDR) protein superfamily. There are many plant proteins at NCBI database (http://www.ncbi.nlm.nih.gov) that are highly similar to HER2, but although putatively annotated, their enzymatic functions have not been determined yet. Among the subject sequences with highest similarity with HER2 at UniProt (http://ebi.ac.uk/uniprot), we found only one protein with a confirmed activity. This is a succinic semi aldehyde (SSA) dehydrogenase acetylating enzyme (A4YGN0, SSADH-Acetylating, EC 1.2.1.76) of a thermo-acidophile bacterium, *Metallosphaera sedula* (Berg et al. 2007), which has 42% aa identity with HER2 (Fig. S3). The reaction catalyzed by this enzyme is as follows: *Succinate semialdehyde (SSA)* + *Coenzyme A* + *NADP* ^+^ *↔ succinyl-CoA* + *NADPH*. However, the SSADH-Acetylating activity has not been reported yet in plants, accordingly to Plant Metabolic Network (PMN, http://www.plantcyc.org) and MetaCyc (http://www.metacyc.org).

**Fig. 5:**
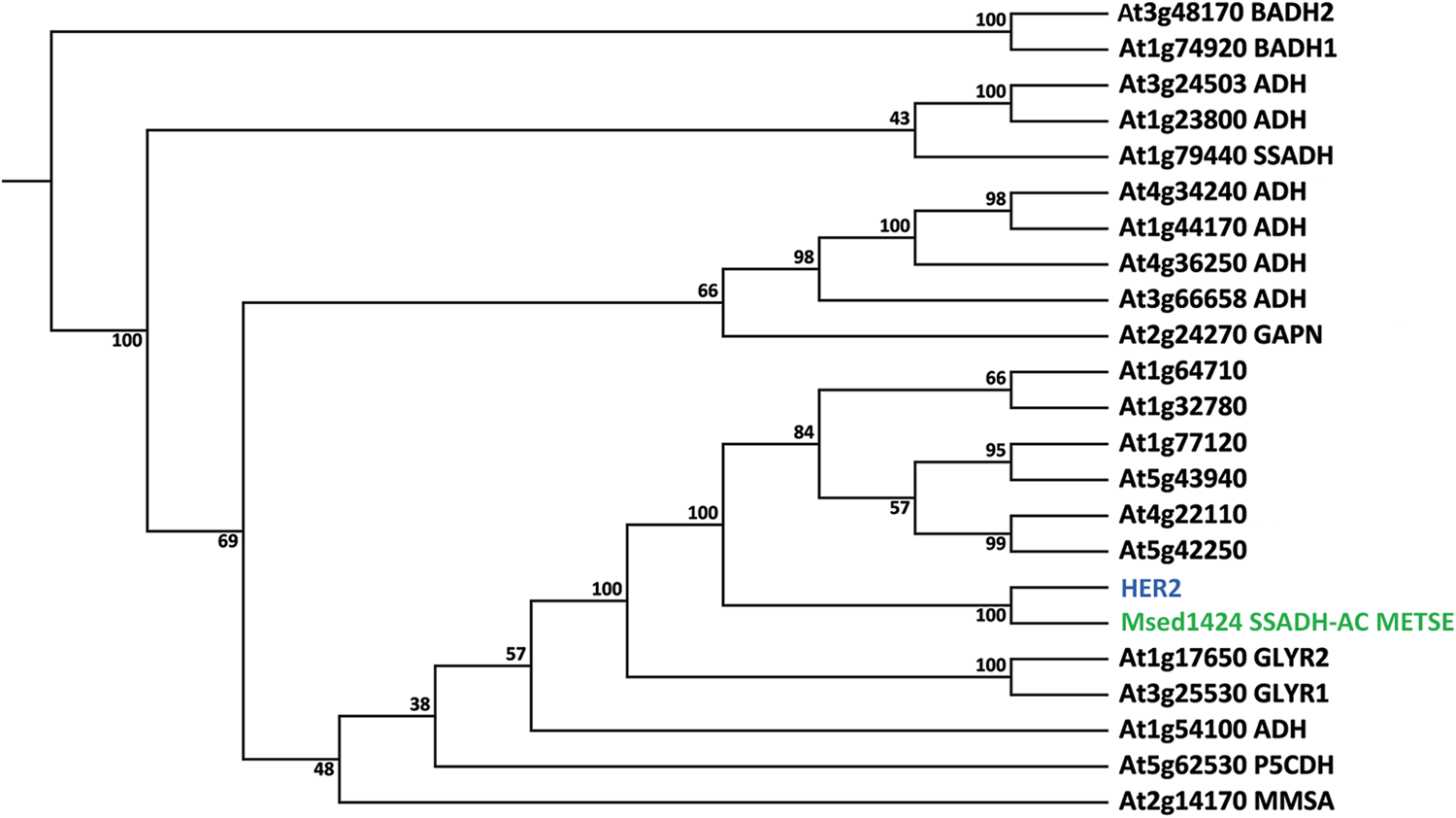
Neighbor joining phylogenetic tree of amino acid sequences of the *A. thaliana* ADH family, AtGLYR1 and AtGLYR2, HER2 (blue) and SSADH-Acetylating A4YGN0 from *M. sedula* (green). ADH, BADH, betaine-aldehyde dehydrogenase; GAPN, non-phosphorylating glyceraldehyde-3-phosphate dehydrogenase; MMSA, methylmalonyl semialdehyde dehydrogenase; P5CDH, D1-pyrroline-5-carboxylate dehydrogenase; SSADH, succinic semialdehyde dehydrogenase; GLYR, glyoxylate reductase; SSADH-AC, succinic semialdehyde dehydrogenase acetylating. At1g64170, At1g32780, At1g77120, At5g43940, At4g22110 and At5g42250 are the six best hits with HER2 as a query against the *Arabidopsis* protein database (http://www.arabidopsis.org). The tree was generated with CLC Main Workbench 6.8.4 (CLC Bio, clcbio.com), using the neighbor joining algorithm with 100 bootstrap simulations

Given the predicted mitochondrial localization, the 42% sequence similarity with an enzyme that uses SSA as a substrate and the annotation as an ADH, we decided to use the ADH and SSADH amino acid sequences of *A. thaliana* (Bouche et al. 2003; Kirch et al. 2004) to calculate a phylogenetic tree (Fig. 5), in order to gain information about the putative function of HER2. We also included AtGLYR1 and AtGLYR2 because they are cytosolic and mitochondrial oxidoreductases, respectively, involved in the conversion of SSA to γ-hydroxybutyrate (GHB) (Allan et al. 2008), plus the amino acid sequences of the top six hits of a BLAST analysis of HER2 against the Arabidopsis protein database (http://www.arabidopsis.org). The phylogenetic tree shows that the HER2 protein and the SSADH-Acetylating protein of *M. sedula* form a separate subcluster (Fig. 5) thus providing little additional evidence about the putative enzymatic activity of HER2. Nevertheless, we produced purified soluble recombinant HER2 protein with a N-terminal GST tag (see Fig. S4 in Supplementary Material) and assayed for SSADH-Acetylating activity (and the reverse reaction) that we could not detect, nor any succinate dehydrogenase (SSADH) activity. Furthermore we tested whether the recombinant GST-HER2 protein could use *E*-2-hexenal or *Z*-3-hexenal as a substrate. This was not the case, unlike the ADH from yeast that was taken along as a control (Fig. S5).

Since the similarity with the SSADH-acetylating enzyme indicates that SSA, an intermediate in the GABA shunt (Ludewig et al. 2008), is a putative substrate of HER2, we investigated whether GABA, the precursor of SSA, accumulated in the *her2* mutant. Still, the levels of GABA in *her2* under unstressed conditions are similarly low as in the wt, while GABA levels in the GABA-transaminase mutant *her1*, which is unable to make SSA from GABA, are much higher as we previously published (Mirabella et al. 2008) (see Fig. S6 in Supplementary Material). This shows that the *her2* mutant does not accumulate GABA unlike the *her1* mutant. Since the *her2* phenotype in soil is not dwarfed like the *ssadh* mutant which accumulates SSA and GHB (Ludewig et al. 2008), we did not measure those two metabolites.

### *E*-2-hexenal changes the redox status of the mitochondria

Since HER2 is predicted to be a mitochondrial oxidoreductase, thus putatively involved in redox reactions, we set out to determine whether HER2 is important for conditions that involve redox changes. To do so we used a redox-sensitive GFP2 (roGFP2; (Meyer et al. 2007)) to monitor the redox state of the cell and in particular, we decided to check both mitochondria and cytosol. We thus transformed wildtype Col-0 and *her2* with constructs driving constitutive expression of *roGFP2* (Ubiquitin10 promoter) and targeting roGFP2 either to the cytosol or mitochondria. In order to confirm the reliability of roGFP2 reporter we sought to measure the fluorescence lifetime in 4 day-old seedlings treated for 30 min with 20 mM hydrogen peroxide (H_2_O_2_) (Wierer et al. 2012). The treatment was performed on seedlings with the reporter expressed in the cytosol or in the mitochondria.

**Fig. 6:**
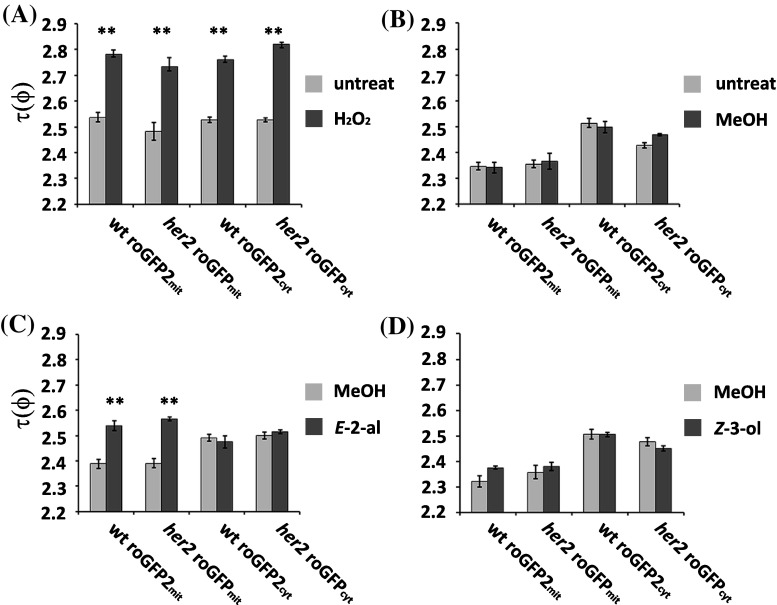
Fluorescence lifetime measurement of roGFP2. The treatment was performed on *her2* seedlings with the reporter expressed in the cytosol (*her2* roGFP2_cyt_) or in the mitochondria (*her2* roGFP_mit_) and on Col-0 seedlings with the reporter expressed in the cytosol (wt roGFP2_cyt_) or in the mitochondria (wt roGFP_mit_) under different conditions: **a** 30 min 20 mM H_2_O_2_ and untreated control, **b** 1 h MeOH and untreated control, **c** 1 h MeOH or 0.3 μM aerial *E*-2-hexenal, **d** 1 h MeOH or 0.3 μM aerial *Z*-3-hexenol. Every experiment was repeated at least three times with similar results

The fluorescence lifetime τ(φ) was measured in the root tip of seedlings as an indication of the oxidation state of the redox sensitive reporter, roGFP2. This reporter has disulphide bonds that react to the redox state of the cell, which results in an increase of the τ(φ) in an augmented oxidized environment (Meyer et al. 2007; Wierer et al. 2012). Figure 6a shows that τ(φ) of the reporter in H_2_O_2_ treated seedlings is statistically significantly higher (P < 0.01, *t*-test) than τ(φ) of untreated seedlings for both the mitochondrial and cytosolic compartments as has been reported for an oxidative treatment (Wierer et al. 2012). No differences were found between *her2* and wt. We then tested methanol since we dissolve GLVs in it. As it is shown in Fig. 6b, MeOH has no effect on τ(φ) in all plant lines. To assess further if the *her2* mutation has an effect on the cellular redox state during GLV perception, we treated the seedlings for 1 h with two different GLVs, 0.3 µM aerial *E*-2-hexenal (Fig. 6c) or 0.3 µM aerial *Z*-3-hexenol (Fig. 6d) because this is one of the most abundantly produced GLVs by *Arabidopsis* (Matsui et al. 2012) or MeOH as negative control.

As shown in Fig. 6d *Z-3*-hexenol had no effect on the τ(φ) of the roGFP2_mit_ expressed in *her2* and wt plants while the *E*-2-hexenal increased τ(φ) significantly (P < 0.01, *t*-Test) (Fig. 6c). Even though we did not see differences between the *her2* mutant and wt, we, interestingly, found that *E*-2-hexenal influenced the redox status in the mitochondria and not in the cytosol. Thus part of the redox events upon *E*-2-hexenal perception takes specifically place in the mitochondria.

## Discussion

*E*-2-hexenal was identified more than 100 years ago as a volatile that is produced by plants (Curtius and Franzen 1914). It has received attention for its ability to induce defense-related responses in plants, but the mechanisms by which plants respond to *E*-2-hexenal, as well as the signaling pathways involved in these responses, remain largely unknown. As an approach to address this we isolated *E*-2-hexenal response mutants (*her* mutants). The characterization of these mutants might help to elucidate the signaling pathways induced by this C6-aldehyde, as *her* mutants are the only mutants to be affected in a specific physiological response to *E*-2-hexenal. Although Col-0 is a natural *hpl* mutant with impaired C6-volatile production, the *her* mutants were isolated in this ecotype because we wanted to exclude the possibility to isolate mutants with altered responses to *E*-2-hexenal associated with altered *E*-2-hexenal production.

The mutation in the *her2* mutant was mapped in the gene At5g63620 (Fig. 1), which is putatively assigned as a member of the ADH family. The genotype-phenotype correlation was first confirmed by the phenotype of a SALK line with a T-DNA insertion in the 1st exon of *HER2* resulting in strongly reduced transcript levels (Figs. 1, S2). Secondly, overexpressing of HER2 (Fig. 2) resulted in a stronger sensitivity to *E*-2-hexenal, as measured by the root growth assay (Fig. 3). Thus this firmly establishes the genotype-phenotype relation.

When HER2 was used as a query for a non-redundant database of protein sequences (UniProt), the only protein with a reported function that shared similarity was a succinic semialdehyde dehydrogenase acylating (SSADH-Acylating) of a thermophile archaea, *M. sedula*. This bacterial SSADH-Acetylating shares 42% identity with HER2 (see Fig. S3 in Supplementary Material) and catalyzes the reduction of succinate semialdehyde (SSA) to succinyl-CoA (Berg et al. 2007; Kockelkorn and Fuchs 2009). Succinate semialdehyde (SSA) is a toxic molecule and its accumulation negatively affects plant growth (Ludewig et al. 2008). Since the *her2* phenotype in soil is not dwarfed as the *ssadh* mutant we can exclude that HER2 accumulates SSA. Conversely, if HER2 would convert SSA to succinyl-CoA, SSA would probably also not accumulate in the *ssadh* mutant (Ludewig et al. 2008). Thus this activity is unlikely and we were also unable to measure it with recombinant HER2 protein. We also tested if HER2 has ADH activity with *Z*-3-hexenal or *E*-2-hexenal as subtrate and NADH or NADPH as cofactors but were unable to establish any activity (see Fig. S5 in Supplementary Material). This would also be remarkable since it would mean that abolishing this ADH activity in the *her2* mutant would actually lead to less conversion of the reactive *E*-2-hexenal to the less reactive *E*-2-hexenol. In fact, *E*-2-hexenol treatment of seedlings does not lead to the inhibition of root growth (Mirabella et al. 2008). Thus the enzymatic activity of HER2 remains to be determined, which is not an easy task. One observation in *her2* seedlings is that root growth is slightly but significantly slower than in wildtype *Arabidopsis* under control conditions (Fig. 1a), indicating that this could be, beside the *E*-2-hexenal response, another phenotype for this mutant.

To investigate the role of HER2, predicted to be an oxidoreductase, in the response to *E*-2-hexenal in a wider context we designed a set of experiments to determine the redox status in the cytosol and mitochondria *in planta*. RES are thought to have an impact on redox signaling in plants (Mueller and Berger 2009), as they do in humans (Zhang and Xiang 2016). We used a redox sensitive GFP (roGFP2; (Meyer et al. 2007), ectopically expressed in wt and *her2* plants, and measured the fluorescence lifetime with FLIM. This technique enables the measurement at a single excitation and emission wavelength and not two wavelengths as needed with confocal laser scanning microscopy (Avezov et al. 2013). It is also photobleaching independent (Nakabayashi et al. 2008) and, with this roGFP2 reporter, it has a twofold-higher sensitivity than confocal microscopy (Meyer et al. 2007; Wierer et al. 2012).

We performed our measurements in the root since it is the organ where the growth inhibition occurs upon *E*-2-hexenal treatment, with chlorophyll conveniently being absent, with in silico analysis showing high *HER2* expression in root tissues (http://www.genevestigator.com). Indeed, 20 mM H_2_O_2_ treatment for half an hour, the positive control, was enough to see roGFP2 become oxidized (Wierer et al. 2012), both in the cytosol and mitochondria (Fig. 6). Next we used volatile treatments under similar conditions as we used to test the root growth phenotype. Methanol, in which *E*-2-hexenal is dissolved, did not change the fluorescence lifetime τ(ϕ) but we noticed that the roGFP2 in the cytosol had a slightly higher basal τ(ϕ) than in the mitochondria. Interestingly, we found that *E*-2-hexenal increased τ(ϕ) in the mitochondria but not in the cytosol indicating that this aldehyde affects specifically the redox status of the mitochondria. This change in redox potential in the mitochondria was not obtained with *Z*-3-hexenol, indicating some sort of specificity in the response. Thus this finding adds a piece to the puzzle of the *E*-2-hexenal signaling pathway, i.e. redox changes specifically taking place in the mitochondria. This also addresses an aspect of the selectivity problem regarding redox-signaling that has been raised for electrophile species (Mueller and Berger 2009): clearly H_2_O_2_ affects the redox status in both the cytosol and mitochondria but *E*-2-hexenal only in the mitochondria. Thus differences between the transcriptomes induced by H_2_O_2_ and the plastidial RES phytoprostane-A1 (Mueller and Berger 2009) or by *E*-2-hexenal (Mirabella et al. 2015) and phytoprostane-A1 (Mueller et al. 2008) could be partly explained by organelle specific redox changes. It is interesting to note that both HER1 (Mirabella et al. 2008) and HER2 are localized in mitochondria suggesting that this organelle is rather important for *E*-2-hexenal signaling. The mitochondria have highly evolved redox processes in relation to respiration and thus maintain a tight redox homeostatis for functioning (Schwarzlander and Finkemeier 2013), and changes in the redox status of the mitochondria are signaled to the cell (Geigenberger and Fernie 2014; Huang et al. 2016; Schwarzlander and Finkemeier 2013). Interestingly, the RES 4-hydroxy-2-nonenal (HNE) can act as a signaling molecule in plant mitochondria (Schwarzlander and Finkemeier 2013) in which it can also modify mitochondrial proteins (Winger et al. 2007). It is conceivable that the redox changes upon *E*-2-hexenal perception occur through activation of the GABA shunt (Mirabella et al. 2015) which changes the NADH/NAD ratio (Allan et al. 2008). It is known that there is a tight functional link between the GABA shunt and the TCA cycle in plants (Michaeli et al. 2011; Studart-Guimaraes et al. 2007). Knocking down the mitochondrial GABA transporter results in higher succinate levels and, vice versa, knocking down succinate CoA ligase results in higher GABA levels. Interestingly, ascorbate levels increase in *Arabidopsis* lines with the mitochondrial GABA transporter knocked down (Michaeli et al. 2011), indicating that the redox state is influenced. Thus together with the recent observation that imbalances in succinate have an effect on mitochondrial ROS (mtROS)(Belt et al. 2017), we speculate that the activation of the GABA shunt by *E*-2-hexenal specifically influences mtROS and not cytosolic ROS through perturbation of succinate homeostasis.

The enzymatic activity of HER2 remains unclear but we speculate it has a minor role in maintaining the succinate balance. Due to its minor role we cannot measure any differences in mtROS with the roGFP that we used. An alternate route for the production of succinate was also suggested (Studart-Guimaraes et al. 2007), but remains elusive. Since the GABA shunt plays an important role in the response to *E*-2-hexenal we thought that perhaps that the flux through this shunt is higher in the *her2* mutant. However, we made a double mutant between *her1*, which lacks GABA transaminase and accumulates GABA, and *her2* but this double mutant does not accumulate more GABA than *her2* disproving this hypothesis (see Fig. S6 in Supplementary Material).

We did not see a difference in the redox status between the wt and *her2* upon *E*-2-hexenal treatment, which could mean that HER2 is not involved in these mitochondrial redox changes. This could also be due to the sensitivity of the roGFP2 or the timing of our measurements after *E*-2-hexenal treatment if differences in redox changes were transient. This explanation is supported by the fact that roGFP2 expressed in the mitochondria has a lower degree of oxidation by H_2_O_2_ than in the cytosol (Schwarzlander et al. 2009) and that the *E*-2-hexenal oxidation of roGFP2 is lower than the one caused by H_2_O_2_ (Fig. 6a, c). Despite the fact that the roGFP2 and FLIM technique have proven to be very useful and reproducible, in the future we could use other redox sensitive markers, such as roGFPiE, which sense the reduction of the redox state in the compartment of interest (Avezov et al. 2013). Another option could be to express the roGFP2 targeted to the mitochondria in HER2 overexpressing lines to achieve a stronger phenotype.

Thus in spite of the fact that the function of HER2 in the mitochondria remains to be determined, as is often the case with forward-genetic screens in Arabidopsis, it is clear from our studies that it is involved in the response to *E*-2-hexenal. Additionally, we have discovered that *E*-2-hexenal changes the redox status of the mitochondria. Very few studies have been done to determine the redox status of an organelle *in planta* and this exciting new technique can now also be used to study the underlying causes (Schnaubelt et al. 2015).

## Electronic supplementary material

Below is the link to the electronic supplementary material.

Supplementary material 1 (PDF 1670 KB)

## References

[CR1] Allan WL, Simpson JP, Clark SM, Shelp BJ (2008). Gamma-hydroxybutyrate accumulation in *Arabidopsis* and tobacco plants is a general response to abiotic stress: putative regulation by redox balance and glyoxylate reductase isoforms. J Exp Bot.

[CR2] Alonso JM (2003). Genome-Wide Insertional Mutagenesis of *Arabidopsis thaliana*. Science.

[CR3] Asai N, Nishioka T, Takabayashi J, Furuichi T (2009). Plant volatiles regulate the activities of Ca2+ -permeable channels and promote cytoplasmic calcium transients in *Arabidopsis* leaf cells. Plant Signal Behav.

[CR4] Avezov E (2013). Lifetime imaging of a fluorescent protein sensor reveals surprising stability of ER thiol redox. J Cell Biol.

[CR5] Belt K, Huang S, Thatcher LF, Casarotto H, Singh KB, Van Aken O, Millar AH (2017). Salicylic acid-dependent plant stress signaling via mitochondrial succinate dehydrogenase.. Plant Physiol.

[CR6] Berg IA, Kockelkorn D, Buckel W, Fuchs G (2007). A 3-hydroxypropionate/4-hydroxybutyrate autotrophic carbon dioxide assimilation pathway in Archaea. Science.

[CR7] Bouche N, Fait A, Bouchez D, Moller SG, Fromm H (2003). Mitochondrial succinic-semialdehyde dehydrogenase of the gamma-aminobutyrate shunt is required to restrict levels of reactive oxygen intermediates in plants. Proc Natl Acad Sci USA.

[CR8] Croft KPC, Juttner  F, Slusarenko AJ (1993). Volatile products of the lipoxygenase pathway evolved from phaseolus vulgaris (L.) leaves lnoculated with Pseudomonas syringae pv phaseolicola. Plant Physiol.

[CR9] Curtius T, Franzen H (1914). Über die chemischen Bestandteile grüner Pflanzen. Über die flüchtigen Bestandteile der Hainbuchenblätter. Justus Liebigs Annalen der Chemie.

[CR10] D’Auria JC, Pichersky E, Schaub A, Hansel A, Gershenzon J (2007). Characterization of a BAHD acyltransferase responsible for producing the green leaf volatile (Z)-3-hexen-1-yl acetate in *Arabidopsis thaliana*. Plant J.

[CR11] Davoine C, Falletti O, Douki T, Iacazio G, Ennar N, Montillet J-L, Triantaphylidès C (2006). Adducts of oxylipin electrophiles to glutathione reflect a 13 specificity of the downstream lipoxygenase pathway in the tobacco hypersensitive response. Plant Physiol.

[CR12] Duran-Flores D, Heil M (2016). Sources of specificity in plant damaged-self recognition. Curr Opin Plant Biol.

[CR13] Engelberth J, Alborn HT, Schmelz EA, Tumlinson JH (2004). Airborne signals prime plants against insect herbivore attack.. Proc Natl Acad Sci USA.

[CR14] Engelberth J, Contreras CF, Dalvi C, Li T, Engelberth M (2013). Early transcriptome analyses of Z-3-hexenol-treated zea mays revealed distinct transcriptional networks and anti-Herbivore defense potential of green leaf volatiles. PLoS ONE.

[CR15] Esterbauer H, Schaur RJ, Zollner H (1991). Chemistry and biochemistry of 4-hydroxynonenal, malonaldehyde and related aldehydes. Free Radic Biol Med.

[CR16] Fall R, Karl T, Hansel A, Jordan A, Lindinger W (1999). Volatile organic compounds emitted after leaf wounding: on-line analysis by proton-transfer-reaction mass spectrometry. J Geophys Res.

[CR17] Farmer EE, Davoine C (2007). Reactive electrophile species. Curr Opin Plant Biol.

[CR18] Farmer EE, Mueller MJ (2013). ROS-mediated lipid peroxidation and RES-activated signaling. Annu Rev Plant Biol.

[CR19] Geigenberger P, Fernie AR (2014). Metabolic control of redox and redox control of metabolism in plants. Antioxid Redox Signal.

[CR20] Gouinguené SP, Turlings TCJ (2002). The effects of abiotic factors on induced volatile emissions in corn. Plants Plant Physiol.

[CR21] Hatanaka A (1993). The biogeneration of green odour by green leaves. Phytochemistry.

[CR22] Heiden AC, Kobel K, Langebartels C, Schuh-Thomas G, Wildt J (2003). Emissions of oxygenated volatile organic compounds from plants part I: emissions from lipoxygenase activity. J Atmos Chem.

[CR23] Hong SM, Bahn SC, Lyu A, Jung HS, Ahn JH (2010). Identification and testing of superior reference genes for a starting pool of transcript normalization in *Arabidopsis*. Plant Cell Physiol.

[CR24] Huang S, Van Aken O, Schwarzlander M, Belt K, Millar AH (2016). The roles of mitochondrial reactive oxygen species in cellular signaling and stress response in plants. Plant Physiol.

[CR25] Jander G, Norris SR, Rounsley SD, Bush DF, Levin IM, Last RL (2002). *Arabidopsis* map-based cloning in the post-genome era. Plant Physiol.

[CR26] Jiang K (2006). Expression and characterization of a redox-sensing green fluorescent protein (reduction-oxidation-sensitive green fluorescent protein) in *Arabidopsis*. Plant Physiol.

[CR27] Kirch H-H, Bartels D, Wei Y, Schnable PS, Wood AJ (2004). The *ALDH* gene superfamily of *Arabidopsis*.. Trends Plant Sci.

[CR28] Kockelkorn D, Fuchs G (2009). Malonic semialdehyde reductase, succinic semialdehyde reductase, and succinyl-coenzyme A reductase from *Metallosphaera sedula*: enzymes of the autotrophic 3-hydroxypropionate/4-hydroxybutyrate cycle in sulfolobales. J Bacteriol.

[CR29] Kunishima M, Yamauchi Y, Mizutani M, Kuse M, Takikawa H, Sugimoto Y (2016). Identification of (Z)-3:(E)-2-hexenal isomerases essential to the production of the leaf aldehyde in plants. J Biol Chem.

[CR30] Ludewig F, Hüser A, Fromm H, Beauclair L, Bouché N (2008). Mutants of GABA transaminase (POP2) suppress the severe phenotype of succinic semialdehyde dehydrogenase ssadh Mutants in *Arabidopsis*. PLoS ONE.

[CR31] Lukowitz W, Gillmor CS, Scheible W-R (2000). Positional cloning in *Arabidopsis*. Why it feels good to have a genome initiative working for you. Plant Physiol.

[CR32] Matsui K (2006). Green leaf volatiles: hydroperoxide lyase pathway of oxylipin metabolism. Curr Opin Plant Biol.

[CR33] Matsui K (2016). A portion of plant airborne communication is endorsed by uptake and metabolism of volatile organic compounds. Curr Opin Plant Biol.

[CR34] Matsui K, Kurishita S, Hisamitsu A, Kajiwara T (2000). A lipid-hydrolysing activity involved in hexenal formation. Biochem Soc Trans.

[CR35] Matsui K, Sugimoto K, Mano Ji, Ozawa R, Takabayashi J (2012). Differential metabolisms of green leaf volatiles in injured and intact parts of a wounded leaf meet distinct ecophysiological requirements. PLoS ONE.

[CR36] Meyer AJ, Brach T (2009). Dynamic redox measurements with redox-sensitive GFP in plants by confocal laser scanning microscopy. Methods Mol Biol.

[CR37] Meyer AJ, Brach T, Marty L, Kreye S, Rouhier N, Jacquot J-P, Hell R (2007). Redox-sensitive GFP in *Arabidopsis thaliana* is a quantitative biosensor for the redox potential of the cellular glutathione redox buffer. Plant J.

[CR38] Michaeli S (2011). A mitochondrial GABA permease connects the GABA shunt and the TCA cycle, and is essential for normal carbon metabolism. Plant J.

[CR39] Mirabella R, Rauwerda H, Struys EA, Jakobs C, Triantaphylides C, Haring MA, Schuurink RC (2008). The Arabidopsis her1 mutant implicates GABA in E-2-hexenal responsiveness. Plant J.

[CR40] Mirabella R (2015). WRKY40 and WRKY6 act downstream of the green leaf volatile E-2-hexenal in Arabidopsis. Plant J.

[CR41] Mochizuki S, Sugimoto K, Koeduka T, Matsui K (2016). *Arabidopsis* lipoxygenase 2 is essential for formation of green leaf volatiles and five-carbon volatiles. FEBS Lett.

[CR42] Mueller MJ, Berger S (2009). Reactive electrophilic oxylipins: pattern recognition signalling. Phytochemistry.

[CR43] Mueller S, Hilbert B, Dueckershoff K, Roitsch T, Krischke M, Mueller MJ, Berger S (2008). General detoxification and stress responses are mediated by oxidized lipids through TGA transcription factors in *Arabidopsis*. The Plant Cell.

[CR100] Nakabayashi T, Nagao I, Kinjo M, Aoki Y, Tanaka M, Ohta N (2008). Stress-induced environmental changes in a single cell as revealed by fluorescence lifetime imaging. Photochem Photobiol Sci.

[CR44] Nelson BK, Cai X, Nebenfuhr A (2007). A multicolored set of in vivo organelle markers for co-localization studies in *Arabidopsis* and other plants. Plant J.

[CR45] Park DH (2010). Mutations in γ-aminobutyric acid (GABA) transaminase genes in plants or Pseudomonas syringae reduce bacterial virulence. Plant J.

[CR101] Pfaffl MW (2001). A new mathematical model for relative quantification in real-time RT-PCR. Nucleic Acids Res.

[CR46] Scala A, Allmann S, Mirabella R, Haring MA, Schuurink RC (2013). Green leaf volatiles: a plant’s multifunctional weapon against herbivores and pathogens. Int J Mol Sci.

[CR47] Schnaubelt D (2015). Low glutathione regulates gene expression and the redox potentials of the nucleus and cytosol in *Arabidopsis thaliana*. Plant Cell Environ.

[CR48] Schwarzlander M, Finkemeier I (2013). Mitochondrial energy and redox signaling in plants. Antioxid Redox Signal.

[CR49] Schwarzlander M, Fricker MD, Sweetlove LJ (2009). Monitoring the in vivo redox state of plant mitochondria: effect of respiratory inhibitors, abiotic stress and assessment of recovery from oxidative challenge. Biochim Biophys Acta.

[CR50] Shiojiri K, Takabayashi J, Yano S, Takafuji A (2000). Flight response of parasitoids toward plant-herbivore complexes: a comparative study of two parasitoid-herbivore systems on cabbage plants. Appl Entomol Zool.

[CR51] Shiojiri K (2006). Changing green leaf volatile biosynthesis in plants: an approach for improving plant resistance against both herbivores and pathogens. Proc Natl Acad Sci USA.

[CR52] Staswick PE, Su W, Howell SH (1992). Methyl jasmonate inhibition of root growth and induction of a leaf protein are decreased in an *Arabidopsis thaliana* mutant. Proc Natl Acad Sci USA.

[CR53] Studart-Guimaraes C, Fait A, Nunes-Nesi A, Carrari F, Usadel B, Fernie AR (2007). Reduced expression of succinyl-coenzyme A ligase can be compensated for by up-regulation of the gamma-aminobutyrate shunt in illuminated tomato leaves. Plant Physiol.

[CR54] Sugimoto K (2014). Intake and transformation to a glycoside of (Z)-3-hexenol from infested neighbors reveals a mode of plant odor reception and defense. Proc Natl Acad Sci USA.

[CR55] Sugimoto K, Matsui K, Takabayashi J (2015). Conversion of volatile alcohols into their glucosides in *Arabidopsis*. Commun Integr Biol.

[CR56] Turlings TC, Loughrin JH, McCall PJ, Rose US, Lewis WJ, Tumlinson JH (1995). How caterpillar-damaged plants protect themselves by attracting parasitic wasps. Proc Natl Acad Sci USA.

[CR57] van Munster EB, Gadella TW (2004). Suppression of photobleaching-induced artifacts in frequency-domain FLIM by permutation of the recording order. Cytometry A.

[CR58] van Munster EB, Gadella TW (2005). Fluorescence lifetime imaging microscopy (FLIM). Adv Biochem Eng Biotechnol.

[CR59] Vermeer JE, Van Munster EB, Vischer NO, Gadella TW (2004). Probing plasma membrane microdomains in cowpea protoplasts using lipidated GFP-fusion proteins and multimode FRET microscopy. J Microsc.

[CR60] Vermeer JE, Thole JM, Goedhart J, Nielsen E, Munnik T, Gadella TW (2009). Imaging phosphatidylinositol 4-phosphate dynamics in living plant cells. Plant J.

[CR61] Weigel D, Glazebrook J (2006). EMS mutagenesis of *Arabidopsis* seed. Cold Spring Harb Protoc.

[CR62] Wierer S (2012). Determination of the in vivo redox potential by one-wavelength spectro-microscopy of roGFP. Anal Bioanal Chem.

[CR63] Winger AM, Taylor NL, Heazlewood JL, Day DA, Millar AH (2007). The Cytotoxic lipid peroxidation product 4-hydroxy-2-nonenal covalently modifies a selective range of proteins linked to respiratory function in plant mitochondria. J Biol Chem.

[CR64] Yamauchi Y, Kunishima M, Mizutani M, Sugimoto Y (2015) Reactive short-chain leaf volatiles act as powerful inducers of abiotic stress-related gene expression. Sci Rep 5:8030. doi:10.1038/srep08030http://www.nature.com/articles/srep08030-supplementary-information Accessed 26 Jan 2015 10.1038/srep08030 PMC4306126 25619826

[CR65] Zebelo SA, Matsui K, Ozawa R, Maffei ME (2012). Plasma membrane potential depolarization and cytosolic calcium flux are early events involved in tomato (Solanum lycopersicon) plant-to-plant communication. Plant Sci.

[CR66] Zhang Y, Xiang Y (2016). Molecular and cellular basis for the unique functioning of Nrf1, an indispensable transcription factor for maintaining cell homoeostasis and organ integrity. Biochem J.

