# Supplementary material for: Forward genetic screens identify a role for the mitochondrial HER2 in E-2-hexenal responsiveness
Source: Plant Mol Biol. 2017 Sep 16;95(4):399–409. doi: 10.1007/s11103-017-0659-8 (PMC5688203; doi:10.1007/s11103-017-0659-8)
Supplement: Supplementary file 1 — Supplementary material 1 (PDF 1670 KB) [file 11103_2017_659_MOESM1_ESM.pdf]

Article title: Forward genetic screens identify a role for the mitochondrial HER2 in *E*-2-hexenal responsiveness

Authors: Scala, Alessandra; Mirabella, Rossana; Goedhart, Joachim; de Vries, Michel; Haring, Michel A.; Schuurink, Robert C.

The following supporting information is available for this paper:

Fig. S1. Chromosome 5 markers and their respective positions in Megabases (Mb).

Fig. S2. *HER2* gene expression in Salk T-DNA lines.

Fig. S3. Alignment of amino acid sequences of HER2, her2 and SSADH-Acetylating enzyme of *Metallosphaera sedula*.

Fig. S4. Western blot with anti-GST antibodies for GST-HER2 purified from *E.coli* .

Fig. S5. Enzymatic activity assays of GST-HER2.

Fig. S6. γ-amino butyric acid (GABA) levels in leaves of wild type, *her1, her2* and the *her1xher2* double mutant under normal conditions.

Table S1. Primer sequences for the markers used for mapping the point mutation in *her2*, At5g63620.

Method S1. Recombinant *HER2* protein purification and enzymatic assays

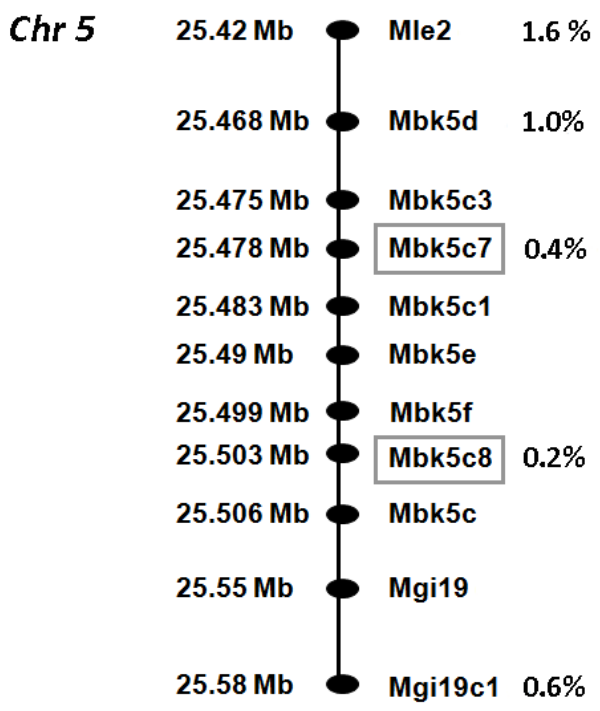

Fig. S1. Chromosome 5 markers and their respective positions in Megabases (Mb). The recombination percentage is indicated next to the marker. *HER2* is located between Mbk5c7 and Mbk5c8 markers (grey boxes). Genes present in this region were sequenced and a point mutation was only found in At5g63620.

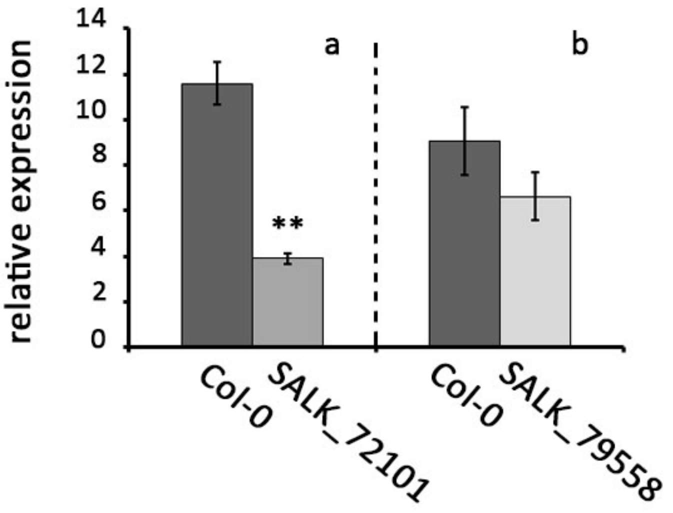

Fig. S2. *HER2* transcript levels in Salk T-DNA lines. *HER2* transcript levels were measured by qRT-PCR in SALK lines 72101 and 79558 and normalized for *SAND* transcript levels. **a** and **b** represent independent experiments, in which the *HER2* expression of each SALK line was compared with those in Col-0. Every bar represents the expression average of 3 different plants (n=3). The *HER2* expression level in SALK_72101 is significantly lower than in Col-0 (T-Test, $p<0.01$).

```
Msed1424_A4YGN0     -------------------------------------------------------MKAAV 5
At5g63620_her2      MAFAPILRRASAVKNLFSLTRLAVGSALSTRSLQTGASGDDSQSMGGYHVSPGGFMRGAV 60
At5g63620_HER2      MAFAPILRRASAVKNLFSLTRLAVGSALSTRSLQTGASGDDSQSMGGYHVSPGGFMRGAV 60
                                                                           *:.**

Msed1424_A4YGN0     LHTYKEPLSIEDVNISQPKAGEVKIKVKATGLCHSDVNVFEGKTPVPPPVVAGHEISGIV 65
At5g63620_her2      YREPNKPLTIEEFHIPRPKSNEILIKTKACGVCHSDLHVMKGEIPFASPCVIGHEITGEV 120
At5g63620_HER2      YREPNKPLTIEEFHIPRPKSNEILIKTKACGVCHSDLHVMKGEIPFASPCVIGHEITGEV 120
                     :  ::**:**:..* :**: *: **.** *:****:.*::*: *.  * * ****:* *

Msed1424_A4YGN0     EEVGPGV-----TRVKPGDRVISAFIHPCGKCGNCVAGKENLCETFSQ-VRLKGVMPDGT 119
At5g63620_her2      VEHGPLTDHKIINRFPIGSRVVGAFIMPCGTCSYCAKGHDDLCEDFFAYNRAKGTLYDGE 180
At5g63620_HER2      VEHGPLTDHKIINRFPIGSRVVGAFIMPCGTCSYCAKGHDDLCEDFFAYNRAKGTLYDGE 180
                     * ** .     .*.  *.**:.*** ***.*. *. *:::*** *    * **.: **

Msed1424_A4YGN0     SRLS--KDGKEIRTFLGGGFAEYAIVGENALTRVPEDMDLEKVAVLGCAGLTGYGAISS- 176
At5g63620_her2      TRLFLRHDDSPVYMYSMGGMAEYCVTPAHGLAPLPESLPYSEFAILGCAVFTAYGAMAHA 240
At5g63620_HER2      TRLFLRHDDSPVYMYSMGGMAEYCVTPAHGLAPLPESLPYSESAILGCAVFTAYGAMAHA 240
                    :**   :* . :  :  **:***.:.  ..*: :**.:  .: *:**** :*.***::

Msed1424_A4YGN0     SKIEPGDTVAVIGVGGVGLSTIQLLRASGAGRIIAVGTKKWKLDRAMELGATDVVNSKEI 236
At5g63620_her2      AEIRPGDSIAVIGIGGVGSSCLQIARAFGASDIIAVDVQDDKLQKAKTLGATHIVNAAKE 300
At5g63620_HER2      AEIRPGDSIAVIGIGGVGSSCLQIARAFGASDIIAVDVQDDKLQKAKTLGATHIVNAAKE 300
                    ::*.***::****:**** * :*: ** **. **** .:. **::*  ****.:**: :

Msed1424_A4YGN0     DPVKAIKEITGGG-PQVVIEAGGNEDTIHMALDSVRIGGKVVLVGLPPATAMIPIRVASI 295
At5g63620_her2      DAVERIREITGGMGVDVAVEALGKPQTFMQCTLSVKDGGKAVMIGLSQAGSVGEIDINRL 360
At5g63620_HER2      DAVERIREITGGMGVDVAVEALGKPQTFMQCTLSVKDGGKAVMIGLSQAGSVGEIDINRL 360
                    * *: *:*****   :*.:** *: :*:  .  **: ***.*::**  * ::  * :  :

Msed1424_A4YGN0     VRGGIEVVGNYGGRPRVDMPKLLELVRQGRYDPSRLVTGRFRLEEINEAVKMLEEGEAIR 355
At5g63620_her2      VRRKVWVIGSYGGRARQDLPKVVKLAESGIFNLTNAVSSKYKFEDAGKAFQDLNEGKIVS 420
At5g63620_HER2      VRRKIKVIGSYGGRARQDLPKVVKLAESGIFNLTNAVSSKYKFEDAGKAFQDLNEGKIVS 420
                    **  : *:*.**** * *:**:::*...* :: :. *:.::::*:  :*.: *:**: :

Msed1424_A4YGN0     SLIIP-- 360
At5g63620_her2      RGVVEIL 427
At5g63620_HER2      RGVVEIL 427
                      ::
```

Fig. S3. Alignment of amino acid sequences of HER2, her2 and SSADH-Acetylating enzyme of *Metallosphaera sedula*. In red are the small and hydrophobic, in blue the acidic, in magenta the basic amino acids, except for H; in green hydroxyl, sulphydryl, amine plus G amino acids. * (asterisk) indicates positions which have a fully conserved residue; : (colon) indicates conservation between amino acids with similar properties (scoring > 0.5 in the Gonnet PAM 250 matrix); . (period) indicates conservation between amino acids with of weakly similar properties (scoring =< 0.5 and > 0 in in the Gonnet PAM 250 matrix). Black arrow indicates the amino acid substitution from S to F in her2.

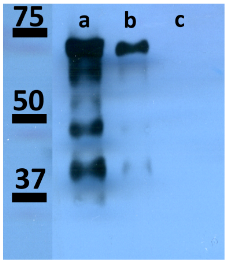

Fig. S4. Western blot with anti-GST antibodies for GST-HER2 purified from *E.coli*. In lanes a, b and c different fractions of the pGEX-KG-HER2 protein purification are shown. Fraction b was used for all experiments. Molecular weight marker is displayed on the left in kDa. HER2-GST is predicted to be 67kDa (HER2 41kDa and GST 26kDa).

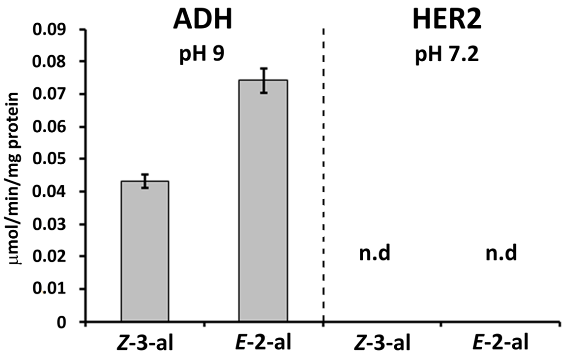

Fig. S5. Enzymatic activity assays of GST-HER2. ADH (yeast alcohol dehydrogenase) is used as positive control. NADH absorbance was measured at 340nm for 200sec and the activity of the enzymes is presented as µmol NAD^+ formed per min per mg protein. Abbreviations: *E*-2-al, *E*-2-hexenal; *Z*-3-al, *Z*-3-hexenal; n.d., no activity detected.

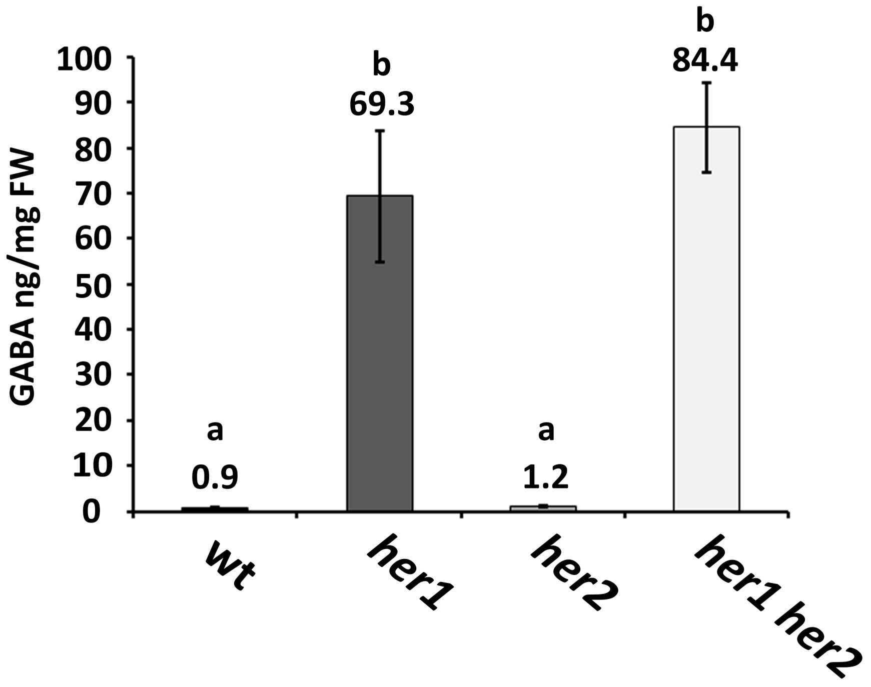

Fig. S6. GABA levels in wild type, *her1* and *her2* leaves under normal conditions. 3 pools from 3 different leaves from 3 weeks old plant were used. Bars represent the mean (± SEM). Bars annotated with different letters indicate significant differences between mutants and Col-0 wild type ($P<0.05$, ANOVA followed by a least significant difference (LSD) post-hoc test).

marker	***Sequence***
ciw9-f	CAGACGTATCAAATGACAAAT
ciw9-r	GACTACTGCTCAAACTATTCG
Mle2-f	GTTAAGTGGTCTCTCATCAG
Mle2-r	CATCACTTATGTCATCACG
Mbk5d-f	CCATGCTTTTGAAAAGCACC
Mbk5d-r	CCGAATACGAAAGTCAAGTC
Mbk5c3-f	GTCCTTCTGATAACCATTCC
Mbk5c3-r	GTGGATCATCTCTTTCACC
Mbk5c7-f	ACGTACATAGGATAGGAGC
Mbk5c7-r	GAAGATCTTCCTGTCTACTG
Mbk5c1 -f	CCACTTGCTCCAACTACGC
Mbk5c1-r	CACCTGTTTCACTGAGGTC
Mbk5e -f	GTGTAAAACTCTTAACTCAAC
Mbk5e-r	ATGTATCTGCTCGAGGAG
Mbk5f -f	TTACACAACAGAGTGAGA
Mbk5f-r	AAGATGTGGAGGACGAGG
Mbk5c8-f	GTGTTACGTGATCTATCTCC
Mbk5c8-r	CACAGTTGTGGTTGGACG
Mgi19-f	GAGAGACAAGATAAGACACC
Mgi19-r	CCAAACTTGACCCTTATACC
MGI19c1-f	AGTACCGGAAGTTGCAGC
MGI19c1-r	TGTAAGTTTCTAGACCTGTC

Table S1. Primer sequences of the markers used for mapping the point mutation in *her2*, At5g63620.

Method S1. Recombinant *HER2* protein purification and enzymatic assays

HER2 was expressed in *E. coli* without its 55 aa signal peptide and with a GST tag at the N-terminus (pGEX-KG vector (Guan & Dixon, 1991)) in order to purify it and to perform *in vitro* activity assays. The construct harbouring *GST-HER2* was transformed to *E. coli* strain BL21-DE3 and expression of the fusion protein was induced using 1 mM IPTG for 6 h at 20°C. The GST fusion protein was purified using affinity chromatography on glutathione agarose as described by (Testerink *et al.*, 2007). Bound protein was eluted from the resin using 20 mM reduced glutathione in 50 mM TRIS-HCl pH 8.0.
The elution fractions were used as input for SDS-PAGE and Western Blot analysis. After separation on an 12% SDS–polyacrylamide gel, proteins were electrotransferred onto a nitrocellulose membrane (GE Healthcare, gelifesciences.com). Immunoblotting was performed with anti-GST monoclonal antibodies (GST(b-14), Tebu-bio, www.tebu-bio.com). An optimal dilution of the antibody at 1:2000 (v/v) in antisera buffer (0.05% (w/v) skim milk, 1× PBS with 0.001% (v/v) Tween-20) was used. Peroxidase-conjugated anti mouse IgG (Fisher, thermofisher.com) diluted at 1:3300 (v/v) was used as secondary antibody for detection with ECL Plus (GE Healthcare, gelifesciences.com).
The fraction containing purified GST-HER2 (lane b, Fig. S4) was used for various enzymatic assays at different pH with the one at pH 7.2 depicted in figure S5. For this figure the reaction was carried out with 100ng protein in 10 mM PIPES pH 7.2 containing 2 mM $ZnCl_2$ in a final volume of 1 ml. As co-factors 0.1 mM NAD^+, NADH, $NADP^+$, or NADPH, and as substrate 0.5 mM *Z*-3-hexenal, *E*-2-hexenal, *Z*-3-hexenol or *E*-2-hexenol were used and the activity was monitored spectrophotometrically at 340 nm. Additionally, 0.5 mM succinic-semialdehyde plus 0.1 mM Co-enzyme A or 0.5 mM sodium succinate were used as substrates. For the positive control yeast ADH (220 units, Sigma) was used in 10 mM Tris.Cl pH 9.0 plus 2 mM $ZnCl_2$.

Guan KL, Dixon JE. 1991. Eukaryotic proteins expressed in Escherichia coli: an improved thrombin cleavage and purification procedure of fusion proteins with glutathione S-transferase. *Anal Biochem* 192(2): 262-267.
Testerink C, Larsen PB, van der Does D, van Himbergen JA, Munnik T. 2007. Phosphatidic acid binds to and inhibits the activity of Arabidopsis CTR1. *J Exp Bot* 58(14): 3905-3914.
